# Cardiopulmonary responses during the cooling and the extracorporeal life support rewarming phases in a porcine model of accidental deep hypothermic cardiac arrest

**DOI:** 10.1186/s13049-016-0283-7

**Published:** 2016-07-08

**Authors:** Guillaume Debaty, Maxime Maignan, Bertrand Perrin, Angélique Brouta, Dorra Guergour, Candice Trocme, Vincent Bach, Stéphane Tanguy, Raphaël Briot

**Affiliations:** Department of Emergency Medicine, SAMU 38, University Hospital of Grenoble, Grenoble, France; University Grenoble Alps/CNRS/TIMC-IMAG UMR 5525/Team PRETA, Grenoble, F-38041 France; Department of Biochemistry Toxicology and Pharmacology, University Hospital of Grenoble, Grenoble, France; Department of cardiac surgery, University Hospital of Grenoble, Grenoble, France

**Keywords:** Deep hypothermic cardiac arrest, Extracorporeal life support, Accidental hypothermia, Cardiac arrest

## Abstract

**Background:**

This study aimed to assess cardiac and pulmonary pathophysiological responses during cooling and extracorporeal life support (ECLS) rewarming in a porcine model of deep hypothermic cardiac arrest (DHCA). In addition, we evaluated whether providing a lower flow rate of ECLS during the rewarming phase might attenuate cardiopulmonary injuries.

**Methods:**

Twenty pigs were cannulated for ECLS, cooled until DHCA occurred and subjected to 30 min of cardiac arrest. In order to assess the physiological impact of ECLS on cardiac output we measured flow in the pulmonary artery using Doppler echocardiography as well as a modified thermodilution technique using the Swan-Ganz catheter (injection site in the right ventricle). The animals were randomized into two groups during rewarming: a group with a low blood flow rate of 1.5 L/min (LF group) and a group with a normal flow rate of 3.0 L/min (NF group). The ECLS temperature was adjusted to 5 °C above the central core. Cardiac output, hemodynamics and pulmonary function parameters were evaluated.

**Results:**

During the cooling phase, cardiac output, heart rhythm and blood pressure decreased continuously. Pulmonary artery pressure tended to increase at 32 °C compared to the initial value (20.2 ± 1.7 mmHg vs. 29.1 ± 5.6 mmHg, *p* = 0.09). During rewarming, arterial blood pressure was higher in the NF than in the LF group at 20° and 25 °C (*p* = 0.003 and 0.05, respectively). After rewarming to 35 °C, cardiac output was 3.9 ± 0.5 L/min in the NF group vs. 2.7 ± 0.5 L/min in LF group (*p* = 0.06). At the end of rewarming under ECLS cardiac output was inversely proportional to the ECLS flow rate. Moreover, the ECLS flow rate did not significantly change pulmonary vascular resistance.

**Discussion:**

Using a newly developed experimental model of DHCA treated by ECLS, we assessed the cardiac and pulmonary pathophysiological response during the cooling phase and the ECLS rewarming phase. Despite lower metabolic need during hypothermia, a low ECLS blood flow rate during rewarming did not improved cardiopulmonary injuries after rewarming.

**Conclusion:**

A low ECLS flow rate during the rewarming phase did not attenuate pulmonary lesions, increased blood lactate level and tended to decrease cardiac output after rewarming. A normal ECLS flow rate did not increase pulmonary vascular resistance compared to a low flow rate. This experimental model on pigs contributes a number of pathophysiological findings relevant to the rewarming strategy for patients who have undergone accidental DHCA.

## Background

Hypothermia by reducing body temperature and therefore the body’s metabolism induces a series a physiological changes to homoeothermic organisms. The initial response to cold exposure is to generate body heat through shivering, a sympathetic response with vasoconstriction, increased oxygen consumption as well as heart and respiratory rate, stroke volume and cardiac output [1]. After this initial phase, the decrease of total body metabolism is proportional to the level of hypothermia. Respiratory rate, as well as heart rate, arterial blood pressure and cardiac output decrease to about 50 % of the normothermic level at 28 °C and about 20 % at 20 °C in laboratory animal and human models [2–4]. Below 28 °C, cardiac arrhythmias are frequent and onset of ventricular fibrillation is the main cause of death in patients suffering accidental hypothermia [1, 5–9].

The cardioprotective and neuroprotective effects as well as the limitation of ischemia/reperfusion injuries mediated by hypothermia are widely used in cardiac surgery and in the treatment of several conditions such as postcardiac arrest syndrome, traumatic brain injury and neonatal anoxia [10].

In case of accidental hypothermia with hemodynamic instability or cardiac arrest, extracorporeal life support (ECLS) is the preferred method of rewarming because it can provide sufficient circulation and oxygenation while the core body temperature can be managed by controlling the blood temperature [6, 9]. Most of the physiological data available on ECLS rewarming were obtained in the context of cardiac surgery.

Femoral arteriovenous cannulation is the method of choice for ECLS rewarming [5, 11]. The venous cannula withdraws blood at the level of the right atrium/vena cava, therefore decreasing right ventricular volume; then oxygenated blood is reinjected in the femoral artery with a retrograde flow. During the ECLS rewarming phase after deep hypothermic cardiac arrest (DHCA) severe cardiopulmonary dysfunction including severe pulmonary edema and adult respiratory distress syndrome have been frequently reported [9, 11]. These complications can be partly explained by ischemia/reperfusion injuries but can also be aggravated by the rewarming strategies [11–13]. On isolated lungs, the flow provided by the pump in the pulmonary artery needs to be reduced compared to theoretical normal cardiac output. Several studies have demonstrated in ex vivo rodent lungs that pulmonary edema was reduced with reduced flow rate [14, 15]. It is recommended to adjust the flow rate to maintain pulmonary artery pressure between 15 and 20 mmHg in order to limit capillary-alveolar membrane lesions [16].

The aim of this study was to assess the impact of ECLS flow rate on cardiac and pulmonary responses during the rewarming phase after DHCA in a porcine model. In addition, our experimental model of accidental hypothermia allowed us to describe the pathophysiological response during the cooling phase until DHCA.

## Methods

### Study design and setting

Animal experiments were carried out in accordance with the relevant French and European Community regulations. All protocols were approved by the Centre de Recherche du Service de Santé des Armées Institutional Animal Care and Research Advisory Committee (Protocol number: 38.04.43).

### Study protocol

#### Preparatory phase

Female Yorkshire pigs, weighing 43.6 ± 0.9 kg, were fasted overnight. Initial sedation was achieved with intramuscular administration of tiletamine and zolazepam (12.5 mg/kg) followed by inhaled isoflurane 2 %. A 20-gauge catheter was placed in an ear vein. The pigs were then curarized with 1.5 mg/kg of intravenous administration of suxamethonium chloride, intubated and mechanically ventilated with isoflurane 2 % to maintain anesthesia. The tidal volume was set at 8 mL/kg with a 50 % inspired oxygen fraction (FiO_2_) and the respiratory rate was adjusted to maintain PaCO_2_ at 40 mmHg. A 5-F Swan-Ganz catheter (Edward Lifesciences, Maurepas, France) was placed in the pulmonary artery, and a 5-F arterial catheter was placed in the right carotid artery.

In addition, a 20-F femoral vein catheter and a 12-F femoral artery catheter (Edwards Lifesciences, Irvine, CA, USA) were inserted using an ultrasound-guided percutaneous approach. Standard cardiopulmonary bypass equipment was used: centrifugal pumps (Cobe Century, Arvada, CO, USA) and a heat exchanger (Sarns Inc., Ann Arbor, MI, USA), a membrane oxygenator (Sorin Group; Munich, Germany), non-heparin-bonded circuit tubing and tank. The tank was primed with 0.5 L of NaCl 0.9 % solution. Heparin (300 units/kg) was injected intravenously just before cannulation.

Surface electrocardiographic tracings were continuously recorded. All hemodynamic data including aortic pressure, pulmonary artery pressure, blood oxygen saturation and end-tidal CO_2_ were continuously monitored and recorded with a digital recording system (BIOPAC MP 150, BIOPAC Systems, Inc., Goleta, CA, USA).

Cardiac outputs were measured with the thermodilution technique using the Swan-Ganz catheter. To allow accurate measurement during ECLS, the injection site of the 5-F Swan-Ganz catheter was located in the right ventricle and the sampling site in the pulmonary artery [17]. An average of at least three measurements of cardiac output that agreed within 10 % was used.

Core temperature was measured using the Swan-Ganz thermistor and with an esophageal probe. Return of spontaneous circulation (ROSC) was defined using the Utstein guidelines for uniform reporting in animal research [18].

Arterial blood gases (Gem 3000, Instrumentation Laboratory, Bedford, MA, USA) were obtained at baseline, then at 30 °C, 25 °C, 20 °C and after 30 min of untreated cardiac arrest at the beginning of the rewarming phase, and then during the rewarming phase at 25 °C, 28 °C, 30 °C, 35 °C and 30 min after 35 °C was reached.

#### Protocol

##### Cooling phase

Hypothermic cardiac arrest was induced using external cold packs (Colpac, Chattanooga group, Chattanooga, TN, USA) associated with ECLS at a very low flow rate of 7 mL/min/kg at 4 °C. To simulate the physiological decrease in respiratory rate during the cooling phase, the isoflurane concentration and respiratory rate were adjusted to the central temperature (respectively, 1.5 % and 15/min <30 ° C, 1 % and 10/min < 25 °C). Cisatracurium besilate 0.15 mg/kg was injected intravenously to induce and maintain curarization. When DHCA occurred (defined by an absence of arterial pressure pulse), ECLS and respiratory support were stopped and the animals were kept for 30 min in untreated cardiac arrest (ischemic period).

##### Rewarming phase

After 30 min of full ischemic state, a total of 20 pigs were distributed according to a preestablished randomization table into two different groups with different ECLS rewarming flow rates (*n* = 10 per group): the LF group with a flow rate of 1.5 L/min (≈35 ml/kg/min) and the NF group with a flow rate of 3 L/min (≈70 mL/kg/min). The temperature in the extracorporeal circuit was adjusted to 5 °C above the central core temperature in all groups. When a central core temperature of 28 °C was reached, external defibrillation was attempted. Two hundred joules external shock was delivered every 2 min until ROSC was achieved. If necessary, after three unsuccessful defibrillations and central temperature above 32 °C, a bolus of 0.5 mg of epinephrine every 4 min and one 150-mg bolus of amiodarone were added until ROSC was achieved. The isoflurane concentration and respiratory rate were adjusted depending on the central temperature with the reverse setting of the cooling phase. After achieving ROSC, the rewarming strategy was continued until the body temperature reached 35 °C. Then final hemodynamic measurements were taken and cardiac function was assessed under different ECLS flow rates: the initial rewarming rate (1.5 or 3 L/min according to randomization group), a standardized low flow rate of 1 L/min and finally the ECLS pump was stopped.

**Fig. 1:**
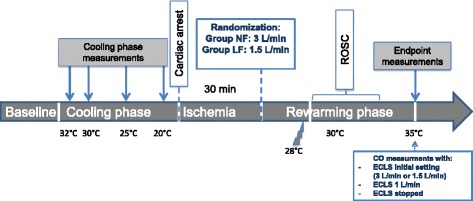
Different phases of the study protocol. ROSC: return of spontaneous circulation, ECLS: extracorporeal life support. * Settings of the ECLS were randomized into two groups: LF, low blood flow rate of 1.5 L/min; NF, normal blood flow rate of 3 L/min. The temperature in the extracorporeal circuit was adjusted to 5 °C above the central core temperature in both groups

The protocol key points are summarized in Fig. 1.

### Measurements

#### Hemodynamic measurements

Except during the rewarming phase before ROSC and at the end of the experiment to access the specific ECLS impact on hemodynamics at different flow rates, all the measurements (cardiac output, systolic and pulmonary arterial pressures) were recorded after stopping the pump of the extracorporeal circuit for 2 min.

Pulmonary vascular resistance (PVR) was calculated as follows:

$$ PVR = \frac{\left( mean\  pulmonary\  arterial\  pressure- mean\  pulmonary\  artery\  wedge\  pressure\right)}{cardiac\  output} $$

#### Doppler echocardiographic evaluation of right cardiac output

**Fig. 2:**
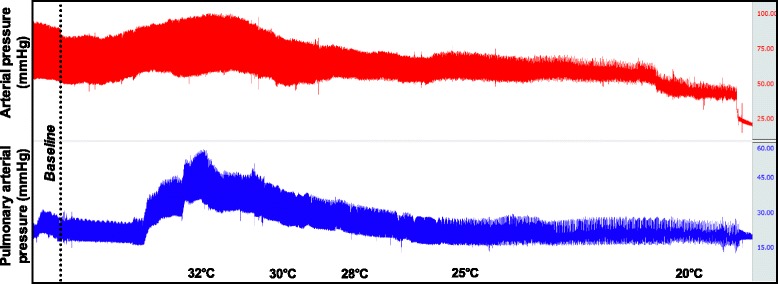
Representative arterial and pulmonary pressure measurement over time during the cooling phase

In order to have an external validation of the modified thermodilution technique, a transthoracic echocardiogram was obtained on the last five pigs included in each group (*n* = 10). Cardiac output measurements were performed at baseline, during the cooling phase at 32 °C, 30 °C, 25 °C and during the rewarming phase at 30 °C and 35 °C with different ECLS flow settings (initial rewarming flow rate (1.5 or 3 L/min), 1 L/min and ECLS stopped). Right cardiac output was computed from the pulmonary artery diameter and the velocity time integral obtained from a parasternal view (Fig. 2).

#### Markers of lung injury

The lung capillary-alveolar permeability to the macromolecule (FITC-Dextran70) was measured by bronchoalveolar lavage (BAL) at the end of the experiment. This method was developed in our laboratory and was previously described and validated in detail [19, 20]. Briefly, we intravenously administered a macromolecular indicator of fluorescein isothiocyanate-labeled dextran (FITC-D70) (Sigma; St. Quentin Fallavier, France). After 25 min, a BAL was performed with 100 mL of a saline solution at 0.9 % with 5 g of albumin. Thirty minutes after administration of the FITC-D70 and 5 min after BAL, we measured FITC-D70 concentrations in both the blood and the BAL fluid, with a fluorescence spectrophotometer (NanoDrop ND-3300 by Labtech; Palaiseau, France). The FITC-D70 transport rate coefficient K_AB_ (min^−1^) (coefficient of permeability of the capillary-alveolar membrane) from blood to alveoli was then estimated by K_AB_ = ([FITC-D70] in BAL/[FITC-D70] in blood)/30.

Distal alveolar fluid clearance (AFC) was measured as previously described. Briefly, following the stabilization period, a catheter (PE 240 tubing; BD; Franklin Lakes, NJ, USA) was passed through a side port in the endobronchial tube into the lung and advanced until gentle resistance was encountered. Then 150 mL of warmed (36 °C) normal saline containing 5 % bovine serum albumin was instilled through the catheter into the airspaces of the lung. After 5 min (T = 0) and 35 min (T = 30 min), samples were removed through the catheter by gentle aspiration. The change in protein concentration at T = 30 min was used to determine the volume of fluid cleared from the airspaces with the following equation:

$$ \mathsf{Distal}\ \mathsf{A}\mathsf{F}\mathsf{C}\ \left(\%/\mathsf{h}\right) = \mathsf{2}\left(\mathsf{1}\ \hbox{-}\ \left(\mathsf{T}\mathsf{0}\ \mathsf{concentration}/\mathsf{T}\mathsf{30}\ \mathsf{concentration}\right)\right) $$

At the end of the experiments, animals were euthanized with intravenous KCL and a segment of the nonlavaged lung was excised and frozen for measurement of the wet-to-dry weight (W/D) ratio. The W/D ratio was determined by drying the lung segments in an oven (60 °C) and then a constant weight was measured over 1 week.

#### Biomarkers

A 3-mL sample of arterial blood was drawn at baseline and at the end of the experiment. Blood samples were centrifuged immediately for 15 min at 2000 *g* at 4 °C. The plasma and BAL samples were then stored at −80 °C until batch-wise analysis. Blood concentrations of pro-inflammatory cytokines, interleukin 1β (IL-1β), IL-6, IL-10 and tumor necrosis factor α (TNF-α) were determined using Biochip Array Technology (Randox Laboratories Ltd, Antrim, United Kingdom).

The blood concentrations of the receptor for advanced glycation end products (RAGEs) were determined using an enzyme-linked immunosorbent assay kit (Cliniscienses, France). RAGEs are a multiligand receptor of the immunoglobulin superfamily of cell surface molecules that acts as a pattern-recognition receptor. RAGEs were initially identified in the lung tissue, which has the highest basal level of expression under normal conditions and is relatively specific to alveolar epithelial cell injury [21, 22].

### Data analysis

Values are presented as mean ± standard error of the mean (SEM). Categorical variables were compared using the Fisher exact test. The Student *t*-test or Mann–Whitney nonparametric test was used to compare quantitative values between groups. To compare biomarker results between groups and between baseline and endpoint measurement, two-way factorial ANOVA for repeated measures was used. Correlation and agreement analyses were done between right cardiac output measured by thermodilution and Doppler echocardiography with the correlation scatter plot, the Pearson correlation method, and the Bland-Altman diagram. All reported *p*-values are two-sided. Significance was defined as *p* < 0.05. Statistical analyses were performed using SPSS software, version 22 (IBM SPSS Statistics, Armonk, NY, USA).

## Results

There were no significant baseline differences between treatment groups (Table 1).

**Table 1:** Characteristics according to randomization group during the cooling and ECLS rewarming phase

		Cooling phase					ECLS rewarming phase			
		Baseline	32	30	25	20	20	25	30	35
LF group	HR, beats/min	100 ± 5	84 ± 9	70 ± 5	55 ± 4	29 ± 9			88 ± 15	124 ± 6
	AP, mmHg	72 ± 7	70 ± 4	44 ± 4	29 ± 2	19 ± 4	27 ± 3*	35 ± 3*	70 ± 9	63 ± 8
	PAP, mmHg	20 ± 2	28 ± 7	24 ± 3	19 ± 3	13 ± 2	8 ± 1	11 ± 2	19 ± 3	33 ± 3
	PVR, dyn · s · cm^−5^	234 ± 50		487 ± 132	515 ± 139				800 ± 419	951 ± 229
	EtCO_2_, mmHg	42 ± 4	27 ± 1	29 ± 1.3	24 ± 2	23 ± 1	11 ± 2	5 ± 1	16 ± 3	33 ± 3
	CO, L/min	4.4 ± .4	3.3 ± .4	2.8 ± .3	2.3 ± .5				1.4 ± .3&	2.7 ± .5
	pH	7.40 ± .02	7.43 ± .02	7.39 ± .03	7.35 ± .04	7.18 ± .02	7.28 ± .04	7.36 ± .06	7.35 ± .05	7.38 ± .04
	PO_2_/FiO_2_, mmHg	449 ± 40	598 ± 69	547 ± 68	549 ± 74	523 ± 191	386 ± 78	934 ± 145	480 ± 118	398 ± 123
	PCO_2_, mmHg	51 ± 3	40 ± 2	46 ± 3	49 ± 5	56 ± 10	54 ± 6	47 ± 6	43 ± 5	37 ± 3
	Serum lactate, mmol/L	2.09 ± .21					7.66 ± 1.55			5.44 ± .54*
	Serum potassium, mmol/L	3.87 ± .15					3.97 ± .13			3.77 ± .17
NF group	HR, beats/min	107 ± 9	80 ± 6	68 ± 6	51 ± 5	25 ± 10			91 ± 9	138 ± 9
	AP, mmHg	68 ± 3	66 ± 5	46 ± 5	28 ± 3	20 ± 1	41 ± 3*	48 ± 3*	64 ± 6	74 ± 7
	PAP, mmHg	21 ± 3	32 ± 15	20 ± 2	16 ± 2	15 ± 6	11 ± 1	12 ± 1	18 ± 3	29 ± 3
	PVR, dyn · s · cm^−5^	287 ± 55		461 ± 54	453 ± 121				480 ± 208	499 ± 93
	EtCO_2_, mmHg	42 ± 4	25 ± 4	26 ± 2	22 ± 2	19 ± 3	11 ± 2	7 ± 2	12 ± 3	30 ± 4
	CO, L/min	4.4 ± .4	3.0 ± .4	2.5 ± .2	1.9 ± .1				1.7 ± .6&	3.9 ± .5
	pH	7.40 ± .02	7.45 ± .14	7.41 ± .04	7.38 ± .03	7.34 ± .01	7.33 ± .03	7.27 ± .03	7.37 ± .03	7.39 ± .03
	PO_2_/FiO_2_, mmHg	433 ± 52	514 ± 164	574 ± 51	684 ± 85	369 ± 45	344 ± 35	783 ± 158	755 ± 103	382 ± 59
	PCO_2_, mmHg	49 ± 3	43 ± 16	43 ± 4	46 ± 3	48 ± 3	52 ± 5	51 ± 3	38 ± 3	33 ± 3
	Serum lactate, mmol/L	2.27 ± .20					5.30 ± .86			3.91 ± .50*
	Serum potassium, mmol/L	3.96 ± .08					3.99 ± .16			3.76 ± .17

Groups: LF/low blood flow rate of 1.5 L/min; NF/normal blood flow rate of 3 L/min. *PaO*_*2*_ */FiO*_*2*_ arterial oxygen pressure/inspired oxygen fraction, *ABP* arterial blood pressure, *PAP* pulmonary arterial pressure, *PVR* pulmonary vascular resistance. Values are presented as mean ± SEM. & measured only on 6 pigs in each group that achieved ROSC at this time point. * *p* < 0.05 compared with the other group

### Cooling phase

**Fig. 3:**
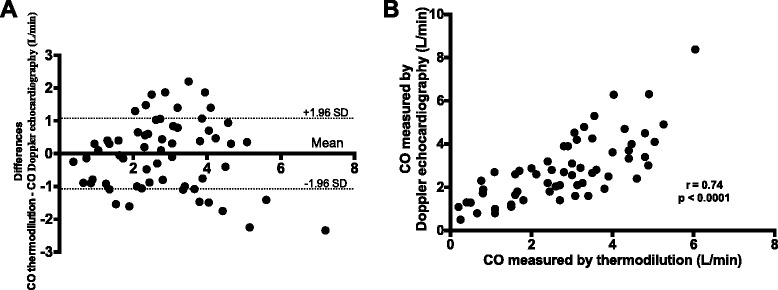
**a** Bland-Altman plot, difference vs. the average right cardiac output measured by thermodilution compared to the Doppler echocardiography method (L/min), **b** Correlation between right cardiac output measured by the thermodilution and the Doppler echocardiography method. CO, cardiac output, r, Pearson correlation coefficient

During the cooling phase, cardiac output, heart rate and mean arterial pressure decreased continuously (*p* < 0.001 for linear trend). The heart rate and arterial pressure were 96 ± 8 % and 117 ± 16 % of basal value at 32°, 68 ± 4 % and 69 ± 8 % at 30°, 54 ± 4 % and 59 ± 7 % at 25 °C and 27 ± 7 % and 24 ± 4 % at 20 °C, respectively (Fig. 2) (*p* < 0.001 for linear trend). The mean pulmonary artery pressure tended to increase at 32 °C compared to baseline (20.2 ± 1.7 mmHg vs. 29.1 ± 5.6 mmHg, *p* = 0.09) and then decreased with core temperature (21.7 ± 1.9 mmHg, 17.4 ± 1.5 mmHg and 13.5 ± 2.4 mmHg at 30, 25 and 20 °C, respectively) (Figs. 2 and 3) (*p* < 0.001 for linear trend). Overall, pulmonary arterial resistance increased with cooling initiation and remained higher than baseline value (262 ± 37, 475 ± 72 and 481 ± 86 dyn · s · cm^−5^ at baseline, 30 and 25 °C, respectively; *p* = 0.03); there was no difference between groups (*p* = 0.34).

Cardiac arrest occurred at 19.4 ± 0.6 °C and there were no differences between groups (*p* = 0.93). Six animals developed ventricular fibrillation, 11 animals presented asystole, and three animals pulseless electrical activity.

At the end of 30 min of untreated cardiac arrest, all pigs were in asystole.

### Rewarming phase

After the beginning of the rewarming phase, the time to reach 30 °C was 43 ± 5 vs. 35 ± 3 min (*p* = 0.19) and the time to reach 35 °C was 105 ± 18 and 74 ± 8 min (*p* = 0.14) in the LF and NF groups, respectively.

Hemodynamics and respiratory parameters during the rewarming phase are presented in Table 1. Mean arterial pressure and mean pulmonary arterial pressure increased continuously during the rewarming phase with core temperature (*p* < 0.001 for linear trend in both groups).

Before ROSC, the mean arterial pressure on ECLS during the rewarming phase was higher in the NF group than in the LF group at 20 °C and 25 °C (*p* = 0.003 and 0.05, respectively).

The mean number of external defibrillations used (3.1 ± 0.7 vs. 2.9 ± 0.6) and adrenaline injected intravenously (0.2 ± 0.08 mg in both groups) to achieve ROSC did not differ between groups (*p* = 0.82 and *p* = 0.72, respectively). All animals were successfully resuscitated: the mean temperature at ROSC was 30.5 ± 0.5 °C in the LF group vs. 30.6 ± 0.7 °C the NF group (*p* = 0.84).

At the end of the rewarming phase, at 35 °C, after the ECLS pump stopped, cardiac output tended to be higher in the NF group than in the LF group: 3.9 ± 0.5 vs. 2.7 ± 0.5 L/min (*p* = 0.06). The serum lactate level at the end of rewarming was higher in the LF group than in the NF group (*p* = 0.04).

### ECLS impact on hemodynamic and doppler echocardiographic measurements

At the end of the rewarming phase, the hemodynamic and respiratory parameters with the ECLS inflow rate set at the initial randomization rate (3 or 1.5 L/min), at 1 L/min or stopped are presented in Table 2.

**Table 2:** Hemodynamic parameters with or without ECLS at 30 °C during the cooling phase and at 35 °C at the end of the rewarming phase

		35 °C ECLS 3 L/min	35 °C ECLS 1.5 L/min	35 °C ECLS 1 L/min	35 °C no ECLS
LF group	HR, beats/min		121 ± 3	117 ± 4	124 ± 6
	AP, mmHg		64 ± 3*	69 ± 4	63 ± 8
	PAP, mmHg		31 ± 6	35 ± 4	33 ± 3
	PVR, dyn · s · cm^−5^		612 ± 65	1268 ± 648	922 ± 411
	EtCO2, mmHg		27 ± 7	31 ± 5	33 ± 3
	Right CO, L/min		2.3 ± .8	2.5 ± .6	3.3 ± .8
	Right CO echo, L/min		3.0 ± 1.1	2.6 ± .7	3.7 ± .8
NF group	HR, beats/min	119 ± 9		128 ± 11	138 ± 9
	AP, mmHg	91 ± 6*		80 ± 8	73 ± 7
	PAP, mmHg	26 ± 4		31 ± 3	29 ± 3
	PVR, dyn · s · cm^−5^	481 ± 74		538 ± 69	456 ± 61
	EtCO2, mmHg	27 ± 3		33 ± 3	30 ± 4
	Right CO, L/min	2.8 ± .8		3.2 ± .6	4.4 ± .5
	Right CO echo, L/min	3.5 ± 1.0		3.4 ± 1.0	4.6 ± 1.0

Groups: LF/low blood flow rate of 1.5 L/min; NF/normal blood flow rate of 3 L/min. *ECLS* extracorporeal life support, *HR* heart rate, *AP* arterial pressure, *PAP* pulmonary arterial pressure, *PVR* pulmonary vascular resistance, *CO* cardiac output

Values are presented as mean ± SEM. Results are presented for the ten pigs with right cardiac output measurement using the Doppler echocardiography method. * *p* < 0.05 compared with the other group

EtCO_2_ (*p* = 0.01 for linear trend), right cardiac output measured by thermodilution (*p* = 0.001 for linear trend) and right cardiac output measured with Doppler echocardiography (*p* = 0.01 for linear trend) were inversely proportional to the ECLS flow rate.

Compared with the thermodilution technique, right cardiac output measurement with Doppler echocardiography had a bias of −0.04 L/min (limit of agreement ± 1.1) (Fig. 3). Including the measurement only when the ECLS pump was running at the end of the rewarming phase, bias was −0.02 L/min (limit of agreement ± 1.1 L/min). The correlation coefficient was r = 0.74 (*p* < 0.001) for all measurements (Fig. 3) and r = 0.72 (*p* = 0.001) for measurements with the ECLS pump running at the end of the rewarming phase.

### FITC-D70 transport rate coefficient K_AB_, wet-to-dry weight ratio and alveolar fluid clearance

The coefficient of permeability of the capillary-alveolar membrane was high in the LF and NF groups: 4.6 ± 1.1 vs. 5.5 ± 1.4 10^−4^.min^−1^, respectively (*p* = 0.63). Distal alveolar fluid clearance was decreased with 7.7 ± 0.3 and 6.4 ± 0.01 % per hour for the LF and NF groups, respectively (*p* = 0.74). The W/D weight ratio did not differ between groups: 6.6 ± 0.1 for the LF group and 6.4 ± 0.3 for the NF group (*p* = 0.51).

### Biomarkers

**Fig. 4:**
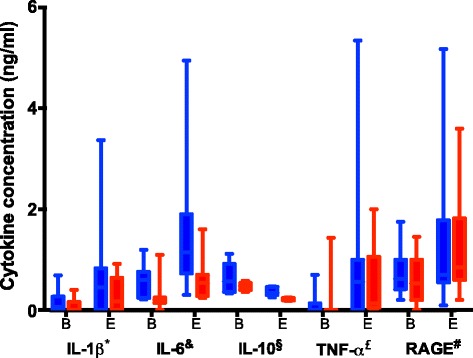
Cytokine concentrations according to group between baseline (B) and endpoint (E) * IL-1β concentration significantly increased between baseline and endpoint (*p* = 0.03), no significant variation between groups (*p* = 0.055). & IL-6 concentration significantly increased between baseline and endpoint (*p* = 0.01); no significant variation between groups (*p* = 0.11). § IL-10 concentration significantly increased between baseline and endpoint (*p* = 0.02); no significant variation between groups (*p* = 0.057). £ TNF-α concentration significantly increased between baseline and endpoint (*p* = 0.03); no significant variation between groups (*p* = 0.23). # RAGE concentration significantly increased between baseline and endpoint (*p* = 0.008); no significant variation between groups (*p* = 0.97)

The serum IL-1β, IL-6, TNF-α and RAGE concentrations at baseline and at the end of the experiment are presented in Fig. 4. There was a significant variation between baseline and endpoint for all cytokines. There was no significant variation of cytokine concentrations between groups (Fig. 4).

## Discussion

Using a newly developed experimental model of deep hypothermic cardiac arrest treated by ECLS [23], we assessed the cardiac and pulmonary pathophysiological response during the cooling phase and the ECLS rewarming phase. A lower ECLS flow rate during the rewarming phase did not attenuate pulmonary lesions, increased the serum lactate level and tended to impair cardiac output after rewarming. Moreover, ECLS at a normal flow rate during rewarming did not increase pulmonary vascular resistance compared to an ECLS at a low flow rate.

In contrast to isolated ex vivo lung models in which a reduced inflow rate in the pulmonary artery was associated with a decrease in the pulmonary edema rate [14, 16], reducing the inflow ECLS rate in our model did not attenuate pulmonary lesions. In a porcine model of ex vivo lungs, pulmonary artery inflow with only 40 % of the theoretical cardiac output value was associated with good viability for 12 h and good results after transplantation [24]. The increased permeability of blood vessels after ischemia/reperfusion injury is increased by any variation of pressure and therefore facilitates liquid extravasation. In ex vivo models, increased inflow pressure led to an immediate increase of pulmonary artery and capillary pressure [16, 24]. This increase put some lung territories under pressure, which had first been collapsed by the protective mechanism associated with hypoxic pulmonary vasoconstriction and hypothermia. In our model, rewarming ECLS generates a systemic blood flow while providing a decrease in right ventricle volume (seen in the present results with the decrease of right cardiac output according to the ECLS flow rate). It has been demonstrated in a human lung model that rewarming after deep hypothermia was associated with vasoconstriction in pulmonary arteries and increased resistance. Nevertheless, after complete rewarming, pulmonary arteries recovered a normal tone similar to that before hypothermia [25]. A decreased volume in the pulmonary circulation under ECLS could limit the increase of PVR and pulmonary artery pressure and thus could provide a benefit to limit pulmonary lesions. In 59 patients, Ruttman et al. found a decrease in the pulmonary edema rate using femorofemoral ECLS compared to cardiopulmonary bypass [11]. While the ROSC rate was similar in both groups, patients treated with cardiopulmonary bypass were weaned faster due to more hemolysis and systemic inflammation. In contrast, using femorofemoral ECLS, circulatory support lasting several days is possible, allowing time for the myocardium and respiratory function to recover before weaning [26, 27].

Since ECLS affects the right and left heart differently, its hemodynamic impact is difficult to analyze without decoupling pulmonary and systemic circulation. In patients with left ventricular function preserved, pressure in the left atrium is decreased by ECLS, while it increases when left ventricular function is altered. Left ventricular overload is facilitated every time that right cardiac output is higher than left cardiac output [28]. In other words, in patients with severe left ventricular dysfunction, ECLS can increase the risk of pulmonary edema [29–32].

For the systemic circulation, higher ECLS flow is related to higher arterial pressure during rewarming. After ROSC, post-charge is increased with higher ECLS flow and global cardiac output depends on ECLS flow and cardiac output [33].

Thermodilution is the reference method to measure cardiac output [34]. However, due to the suction effect of ECLS on right atrial blood, this method is unreliable when ECLS is running. To remedy this problem, we used a modified thermodilution technique validated by Grimbert et al. positioning the injection site of the Swan-Ganz catheter in the right ventricle [17]. They described this method’s good validity albeit a tendency to overestimate right cardiac output in a very low flow state (around 20 % overestimated for right cardiac output measured at 0.75 L/min). Despite lower reliability than the thermodilution technique, cardiac output measurement using Doppler echocardiography has been suitably validated. The reliability of the measurement depends on accurate positioning of the probe to measure the pulmonary artery diameter [34]. The agreement in our results between thermodilution and Doppler echocardiography measurements confirms the inversely proportional relationship between right cardiac output and ECLS flow rate after rewarming after DHCA.

During cooling, the heart rate, arterial pressure, and cardiac output decreased linearly with core temperature, as shown in previous studies [4, 35, 36]. Mean pulmonary arterial pressure increased at the beginning of cooling and PVR values were consistently higher than the initial values. This increase of pulmonary arterial pressure could be related to the low ECLS flow rate we used to facilitate cooling. This effect could be similar to the use of cold saline infusion for cooling induction in targeted temperature management and could give some insight into the increased rate of pulmonary edema described with this cooling technique [37–40].

Despite the potential protective effect of deep hypothermia, in this study ischemia/reperfusion injuries and ECLS rewarming were associated with increased permeability of the capillary-alveolar membrane and substantial variation of proinflammatory cytokines and markers of lung injuries. As compared with reference values in our laboratory, lung capillary-alveolar permeability to the macromolecule and distal alveolar clearance were impaired [20, 22, 41, 42]. There was a trend in our results to lower variation in IL-1β and IL-10 and a decrease in the serum lactate level at the end of rewarming in the NF group.

From a clinical point of view, it is worth noting that if serum potassium concentration is proposed as a triage tool to avoid unnecessary prolonged resuscitation in hypothermic cardiac arrest, in our study the higher potassium concentration at the end of the ischemic period was 5 mmol/L [5, 43]. It is also interesting to remember, as shown in our results, that the usual EtCO_2_ values used to determine the futility of pursuing resuscitation in normothermia have not been validated in the hypothermia context. Because of a lower metabolism, EtCO_2_ decreases proportionally with core temperature [4, 44, 45].

### Limitations

Many variables in that model could have influenced reperfusion injuries (e.g., anesthesia, cooling rate, hypothermia duration, rewarming). However, all animals underwent the same procedure until the beginning of rewarming.

It is possible that hemodynamic parameters were affected by the very low flow rate of the ECLS pump used to facilitate cooling during the cooling phase. Nevertheless, the results in terms of heart rate, arterial pressure and cardiac output are similar to those found in several studies [3, 4, 46, 47]. The rewarming rate in both groups was relatively fast (8.8 °C/h in the low flow group vs. 12.8 °C/h in the normal flow group). As previously observed [23], the main determinant in rewarming rate is the delta of temperature between core and ECLS, it is possible that a more important difference in rewarming rate, a longer rewarming phase or a longer duration of ECLS support could have modified the results.

## Conclusion

We assessed the cardiac and pulmonary pathophysiological response during cooling and ECLS rewarming in a porcine model of DHCA treated with ECLS.

These results suggest that a lower flow rate during the rewarming phase of DHCA did not improve cardiac or pulmonary outcomes. Therefore, the impact of other parameters such as the duration of ECLS rewarming or targeted temperature management could be further evaluated as they affect the prognosis of DHCA patients.

## Abbreviations

AFC, Distal alveolar fluid clearance; BAL, Bronchio alveolar lavage; CPR, cardiopulmonary resuscitation; DHCA, deep hypothermic cardiac arrest; ECLS, extracorporeal life support; ETCO2, end-tidal carbondioxide; FiO_2,_ inspired oxygen fraction; PVR, Pulmonary vascular resistance; RAGE, receptor for advanced glycation end product; ROSC, return of spontaneous circulation; SEM, standard error of the mean.
